# Characterization of DNA Topoisomerase-1 in *Spodoptera exigua* for Toxicity Evaluation of Camptothecin and Hydoxy-Camptothecin

**DOI:** 10.1371/journal.pone.0056458

**Published:** 2013-02-22

**Authors:** Lan Zhang, Dejun Ma, Yanning Zhang, Weizhi He, Jingjing Yang, Chuanren Li, Hongyun Jiang

**Affiliations:** 1 Key Laboratory of Integrated Pest Management in Crops, Ministry of Agriculture, Institute of Plant Protection, Chinese Academy of Agricultural Sciences, Beijing, China; 2 College of Agriculture, Yangtze University, Jingzhou, China; 3 College of Natural Resources and Environment, South China Agricultural University, Guangzhou, China; U. Kentucky, United States of America

## Abstract

Camptothecin (CPT), a plant alkaloid originally isolated from the native Chinese tree, *Camptotheca acuminate*, exerts the toxic effect by targeting eukaryotic DNA topoisomerase 1 (DNA Topo1). Besides as potent anti-cancer agents, CPT and its derivatives are now being explored as potential pesticides for insect control. In this study, we assessed their toxicity to an insect homolog, the Topo1 protein from beet armyworms (*Spodoptera exigua* Hübner), a worldwide pest of many important crops. The *S. exigua Topo1* gene contains an ORF of 2790 base pairs that is predicted to encode a polypeptide of 930 amino acids. The deduced polypeptide exhibits polymorphism at residue sites V420, L530, A653 and T729 (numbered according to human Topo1) among insect species, which are predicted to confer sensitivity to CPT. The DNA relaxation activity of this protein was subsequently examined using a truncated form that contained the residues 337–930 and was expressed in bacteria BL21 cells. The purified protein retained the ability to relax double-stranded DNA and was susceptible to CPT and its derivative hydroxy-camptothecin (HCPT) in a dose-dependent manner. The same inhibitory effect was also found on the native Topo1 extracted from IOZCAS-Spex-II cells, a cell line established from beet armyworms. Additionally, CPT and HCPT treatment reduced the steady accumulation of Topo1 protein despite the increased mRNA expression in response to the treatment. Our studies provide information of the *S*. *exigua* Topo1 gene and its amino acid polymorphism in insects and uncover some clues about potential mechanisms of CPT toxicity against insect pests. These results also are useful for development of more effective Topo1-targeted CPT insecticides in the future.

## Introduction

DNA topoisomerases (DNA Topos), the enzymes that participate in DNA strand breakage and reunion reactions in a series of genetic processes such as DNA replication, transcription and recombination during the cell growth and proliferation, have been a target for cancer therapy [1]. A potent inhibitor of this class of enzymes is camptothecin (CPT), a plant alkaloid isolated originally from the Chinese tree, *Camptotheca acuminate* Decne. CPT functions by binding to and stabilizing the covalent complex of the nicked DNA-Topo1, which prevents DNA re-ligation and therefore causes irreversible DNA break during ongoing DNA and RNA synthesis [2]. Ever since its discovery in 1988, a plethora of studies have demonstrated that inhibition of eukaryotic Topo1 by CPT and its derivatives hinder cell survival and lead to apoptosis [3]–[5]. Due to the remarkable anti-cancer activity, CPT has been developed into a classical anticancer agent [3], [6]. In addition, two of its classic derivatives, irinotecan and topotecan, have been used for treatment of various cancers throughout the world, and meanwhile some CPT analogues are currently used at various stages of clinical trials [7].

Although CPT was not known till 1966 during a discovery screening of natural products for anti-cancer drugs, the crude extract of *C. acuminate* that contains the now-known CPT has been traditionally used to control pests in ancient China for centuries. CPT and its derivatives are now being explored as a class of botanical insecticide in agriculture. CPT is a potent chemosterilant against houseflies [8]. It also exhibits significant genotoxicity to fruit flies (*Drosophila melanogaster* Meigen), which acts through promoting homologous and illegitimate recombination, as well as the whole chromosome loss by clastogenicity, deeply elucidating essential functions of Topo1 in embyrogenesis, oogenesis, larva and pupal growth [9]–[11]. Recently, CPT has been shown to be toxic to several other agricultural pests like brown plant hoppers (*Nilaparvata lugens* Stål), striped rice borers (*Chilo suppressalis* Walker), cabbage aphids (*Brevicoryn brassicae* Linnaeus) and small citrus trypetids (*Bactrocera dorsali* Hendel) [12], [13]. To find new CPT-derived insecticides with improved efficacy and to determine the potential structural factors required for the biological activity of CPT analogues, a series of novel CPT derivatives have been semi-synthesized, and some of them were found toxic to armyworms (*Mythimna separate* Walker) and coconut palm beetles (*Brontispa longissima* Gestro) [14], [15]. In our previous studies, we found that 0.1–30 µM CPT treatment to IOZCAS -Spex-II cells (established from beet armyworms, *Spodoptera exigua* Hübner) induce cell apoptosis [16]. Thus, these previous findings intrigue us to explore CPT as a lead compound of an insect control agent, and to investigate the possibility of exploring a new Topo1-targeted botanical insecticide.

Despite efforts on the toxicity of CPT to agricultural pests, very limited studies have been focused on insect Topo1s except the fruit fly (*D. melanogaster*) [11], [17]–[19], and it has remained yet clear of whether CPT cytotoxicity to insect cells is closely associated with *Topo1* gene expression and Topo1 enzyme activity. Here, we isolated and characterized the beet armyworm *Topo1* gene and evaluated the susceptibility of Topo1 to CPT and its derivative hydroxyl-camptothecin (HCPT). We found that the Topo1 protein, when either purified as a recombinant protein from bacteria or extracted from IOZCAS-Spex-II cells, was susceptible to both CPT and HCPT. Moreover, pretreatment with CPT and HCPT led to reduction in both the enzymatic activity and the steady accumulation of the Topo1 protein in IOZCAS-Spex-II cells despite up-regulation of its mRNA expression in response to the treatment. These results offer important information for understanding CPT-toxicity against insects from the point of adaptive evolution and molecular toxicology and have implications in designing potent Topo1-based pesticides with high-performance and environment safety.

## Results

### Characterization of S. exigua Topo1 Sequence

**Figure 1:**
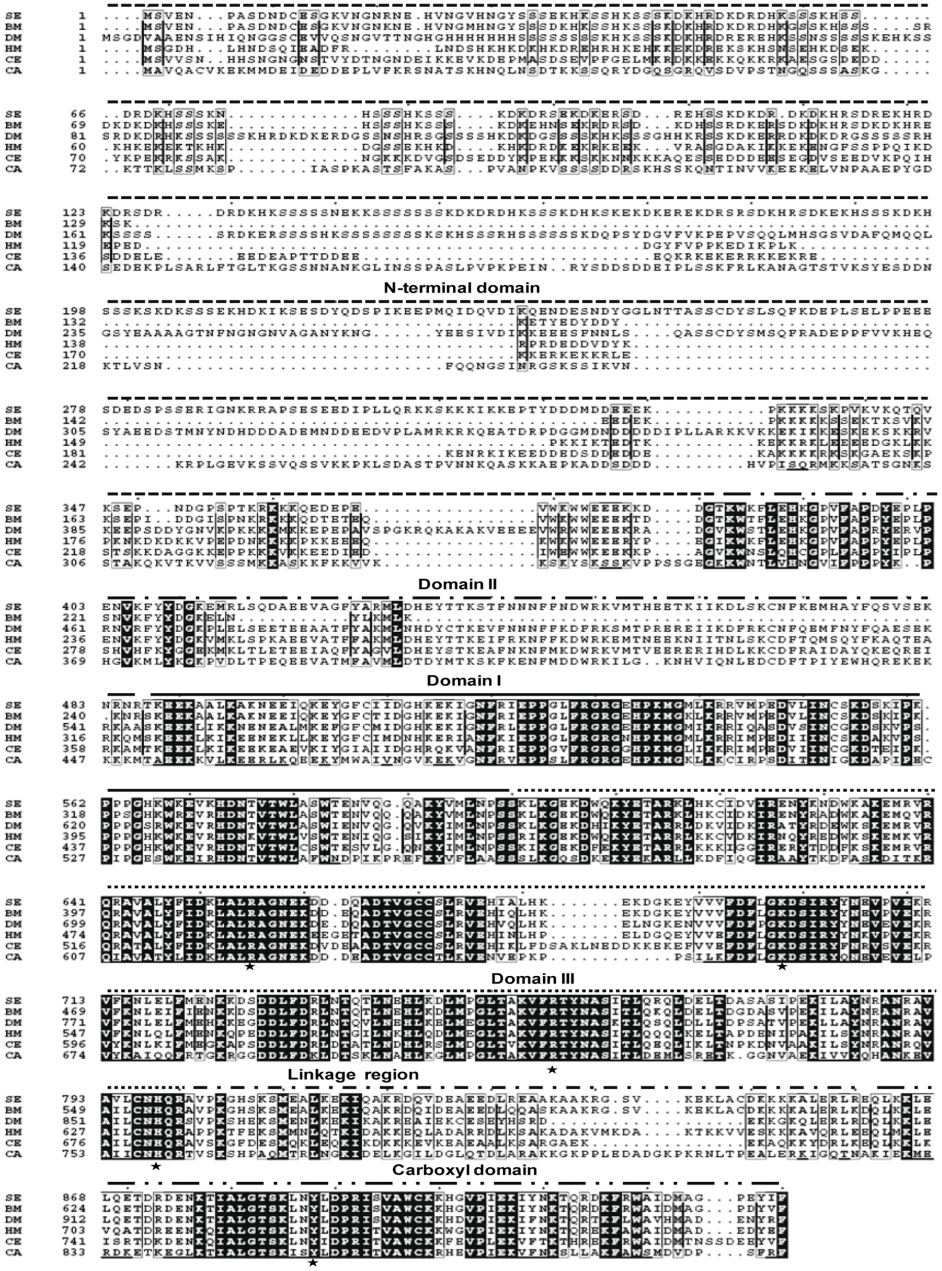
Multiple sequence alignment of Topo1. The amino acid sequences of Topo1 proteins from six representative species were aligned using ClustalX 1.83 with standard parameters and then rendered with ESPript 2.2 for clear illustration. Identical amino acids were highlighted in filled black columns. N-terminus region, aa 1–380; Domain I, aa 486–599; Domain II, aa 381–485; Domain III, aa 600–800; Carboxyl domain, aa 861–931; Linkage region, aa 801–859 (numbered according to human Topo1). The asterisk represents the key residue involved in the catalytic process. Abbreviations: SE, *Spodoptera exigua* (GenBank ID: JN258956); BM, Bo*mbyx mori* (KAIKOGA029083); DM, *Drosophila melanogaster* (GenBank ID: NM078606); HM, *Homo sapiens* (GenBank ID: J03250); CE, *Caenorhabditis elegans* (GenBank ID: NM060936); CA, *Camptotheca acuminate* (GenBank ID: AB372511).

To isolate the *S. exigua Topo1* gene, three sets of degenerate primers targeting the conserved regions of eukaryotic *Topo1* genes were employed to amplify the *Topo1* coding region. The 5′ and 3′ ends were obtained with RACE PCR. The full-length *Topo1* mRNA has 3740 nucleotides in length, encoding an ORF of 2790 nt flanked by a 80 nt 5′ UTR and a 870 nt 3′ UTR. The deduced Topo1 polypeptide has a size of 930 amino acids with a calculated molecular weight of 108 kDa and an estimated pI of 9.47, belonging to the type IB topoisomerases according to phylogenetic analysis. Multiple protein sequence alignment also indicates that *S. exigua* Topo1 exhibits high homology (65.3% identity) with *Bombyx mori* Topo1 and shares 42.6% identity with all species listed in Figure 1. Like its eukaryotic counterparts, the *S. exigua* Topo1 consists of four major regions, namely the N-terminal domain, the core domain, a poorly conserved linker region, and the C-terminal domain [20]. The core and carboxyl domains of *S. exigua* Topo1 are highly conserved in structure organization and contain all previously identified active-site residues (R488, K532, R590 and H632) and the catalytic Y723 (numbered according to human Topo1). In contrast, the N-terminal region is poorly conserved, which has been shown not strictly required for Topo1 catalytic and relaxation functions [20]. The linker region is highly positively charged and flexible, dispensable for Topo1 enzyme activity [21].

**Figure 2:**
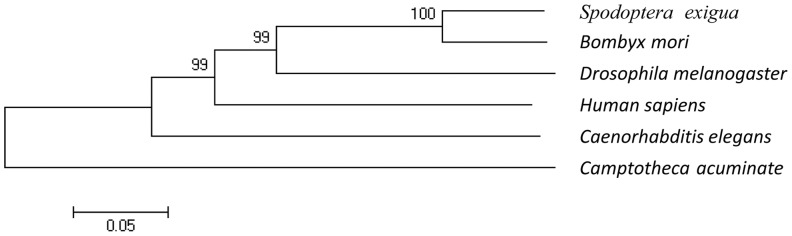
Phylogenetic analysis of Topo1. The amino acid sequences of Topo1 from different spices, including beet armyworm (*S. exigua*), fruit fly (*D. melanogaster*), silkworm (*B. mori*), nematode (*C. elegans*), CPT-producing plant (*C. acuminate*) and human (*H. sapiens*), were aligned with MEGA5.0 program. A phylogenic tree was constructed by the neighbor-joining method with 1,000 replicates. The genetic distance was drawn to scale and the bootstrap value illustrated above the line was marked in numbers.

The phylogenic tree of selected six Topo1s is shown in Figure 2. Six Topo1s from beet armyworm (*S. exigua*), fruit fly (*D. melanogaster*), silkworm (*B. mori*), nematode (*Caenorhabditis elegans*), *C. acuminate* and *Homo sapiens* are split into three divergent clades according to the bootstrap value among those species. The neighbor-joining tree of Topo1s shows that the bootstrap value between insects mentioned above and *H. sapiens* approximate to 99%, which constitutes a large branch. However, the other two clades including *C. elegans* and *C. acuminate* receive none of the branch support in neighbor-joining analyses with no observable bootstrap values. Of note, Topo1s of *S. exigua* and *C. acuminate* share a very weak distance-based relationship in an evolutionary way, which predicts that *S. exigua* Topo1 may be a native CPT sensitive type.

### Amino Acid Polymorphism in Insect Topo1s Related to CPT Sensitivity or Resistance

The amino acid polymorphism of Topo1 has been shown to play an important role in conferring sensitivity or resistance to CPT and its derivatives during evolution. In general, CPT is very toxic to eukaryotic Topo1s except to those from the CPT-producing plants like *C. acuminate*, *Nothapodytes foetida* Wight, and also *Fusarium solani* (INFU/Ca/KF/3, an endophytic microorganism living on *C. acuminate*) [22], [23]. This paradox has been explained by a theory of adaptive coevolution between CPT biosynthesis and CPT-resistant Topo1s derived from Topo1-based mutations in the CPT-producing plants [22]. Three amino acid substitutions, N421K, L530I and N722S (numbered according to human Topo1) found in those CPT-producing plants, have been documented to be associated with the resistance to CPT [22]. Moreover, the same mutations were also detected in some human cancer cells extracted from patients who were tolerant to CPT treatments. Lots of reports have revealed that mutations in Topo1 that affect CPT binding or catalytic process can confer animal cells effective resistance to various extent [24]–[26]. Thus, the polymorphism of Topo1 protein provides useful information for prediction of CPT resistance or sensitivity before performing cellular toxicity tests.

**Figure 3:**
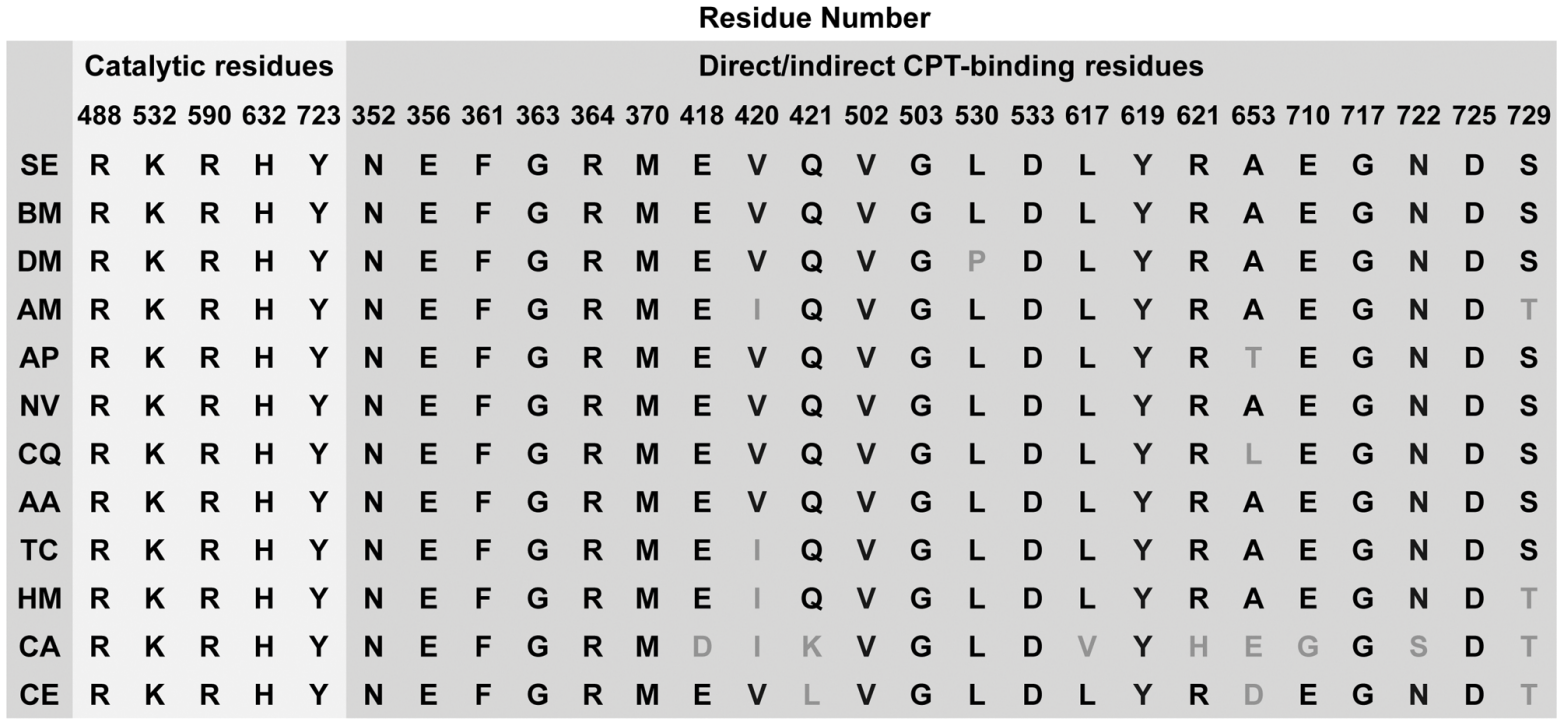
Amino acid polymorphism in Topo1s related to catalytic functions and direct/indirect CPT binding capacity. The five residues essential for catalytic activity are colored white, while the residues involved in binding to CPT are marked gray. The amino acid substitutions were highlighted in light gray. The residues are numbered according to the relative position in human Topo1. Abbreviations: SE, *Spodoptera exigua* (GenBank ID: JN258956); BM, *Bombyx mori* (KAIKOGA029083); DM, *Drosophila melanogaster* (GenBank ID: NM078606); AM, *Apis mellifera* (GenBank ID: XM_396203); AP, *Acyrthosiphon pisum* (GenBank ID: XM_001942991); NV, *Nasonia vitripennis* (GenBank ID: XM_001605054); CQ, *Culex quinquefasciatus* (GenBank ID: XM_001845544); AA, *Aedes aegypti* (GenBank ID: XM_001655563); TC, *Tribolium castaneum* (GenBank ID: XM_966102); HM, *Homo sapiens* (GenBank ID: J03250); CA, *Camptotheca acuminate* (GenBank ID: AB372511); CE, *Caenorhabditis elegans* (GenBank ID: NM060936).

To reveal the amino acid polymorphism of insect Topo1s, we performed sequence alignment analysis. The key residues including R488, K532, R590, H632 and Y723 (numbered according to human Topo1) were found evolutionarily conserved, further supporting their essential role in the nicking-closing reaction. Amino acid substitutions were observed at several positions, including V420, L530, A653 and T729, which may affect the sensitivity of insects to CPT and its derivatives. It has been reported that V420I do not alter sensitivity of CPT-producing plants to CPT, in contrast to previous studies that residue substitutions in amino acids 410–429 confer CPT-resistance in human cancer cells [22], [27]. Unlike T729 in human and plants, S729 exists in every insect listed in Figure 3 except *Apis mellifera*. Other substitutions at position T729 associated with differential CPT resistance have been documented, but T729S has not been mentioned [28], [29].

### Truncated Topo1 Expressed in E. Coli BL21 (DE3) Cells Retained the Same DNA-relaxing Enzyme Activity as Natural Topo1 in *S. exigua*

**Figure 4:**
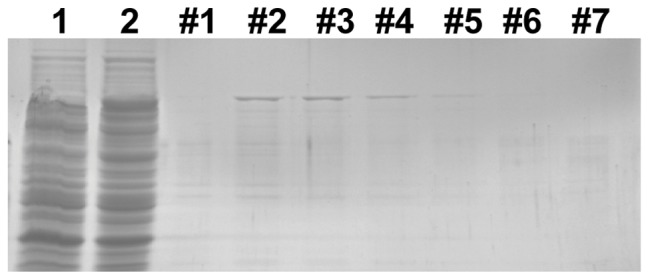
Analysis of purified Topo1 preparations by SDS-PAGE. A truncated form of *S. exigua* Topo1 containing residues 337–930 was expressed as a GST fusion protein in *E. coli* BL21 cells, and purified using the trap column as described in Materials and Methods. Lane 1, cell lyses of bacteria BL21 expressing GST alone; Lane 2, cell lyses of bacteria expressing GST-Topo70; Lane 3–9, serial fractions of eluted Topo1 (#1–7).

The N-terminal domain of Topo1 is highly heterogeneous and has been shown to be not required for the DNA relaxation activity in the case of Human Topo1. As *S. exigua* Topo1 is highly homologous with human Topo1, an N-terminus truncated form containing residues 337–930 of *S. exigua* Topo1 was expressed as a GST fusion protein in *E. coli* BL21 cells to test its enzymatic activity. Single expression of GST served as a negative control. The bacterially expressed truncated Topo1 (named Topo70) was subsequently purified by GSTrap columns and analyzed by SDS-PAGE. As expected, a protein with approximate 97 KDa corresponding to the predicted size of GST-Topo70 was observed on the gel by commassie blue staining (Figure 4). The enzymatic activity of Topo70 *from E. coli* cell lysates was determined by serial two-fold dilutions. The specific activity of Topo70 from purified fraction and crude lysate was 1,797,600 and 344,000 U mg^−1^ pro, respectively (Table 1), suggesting that the truncated Topo1 function as well as the natural Topo1 did in IOZCAS-Spex-II cells.

**Table 1:** Topo1 protein purification and the enzyme activity.

Usage	Gene	Forward primers (5′–3′)	Reverse primers (5′–3′)	Size(bp)
RT-PCR	Topo1	GAACCNCCNGGNYTNTTCMGNG	ATNGCNCANGCNCGRTTNG	770
		AAAAGGATGATGACCAGGCAGAC	CNGGNCCNGCCATRTCDATNGCCCA	805
		CAYAARGGNCCHGTNTTYGCWCCHG	CCAGGAGGTGGTTTAGGTA	530
5′ RACE	Topo1	CGCGGATCCACAGCCTACTGATGATCAGTCGATG	GCATAGAATCCCGCTACTTCCTCAG	1419
3′ RACE	Topo1	GCTGTTCCCAAAGGTCATTCA	CGCGGATCCTCCACTAGTGATTTCACTATAGG	1294
Full-length cDNA	Topo1	ATGAGTGTCGAAAATCCCGCTAGCGA	TTAGAAGATATATTCCGGACCGGCCA	2793
Truncated cDNA	Topo70	GCTCGGATCCAAACCAGTGAAAGTCAAACAAACTCAAGT^a^	CATGTCGACGAAGATATATTCCGGCCCCGCCA^b^	1782
Real-time PCR	Topo1 Beta-actin	AGGTTTGGAAATGGTGGGAAGA TTCCAGCCTTCCTTCTTGGGTAT	CATGATCTAACATTCGAGC GGGAGCGATGATCTTGATCTTGA	198
				213

### CPT and HCPT Exhibited a Dose-dependent Inhibition on the Enzyme Activity of Topo1 Extracted in IOZCAS-Spex-II Cells

**Figure 5:**
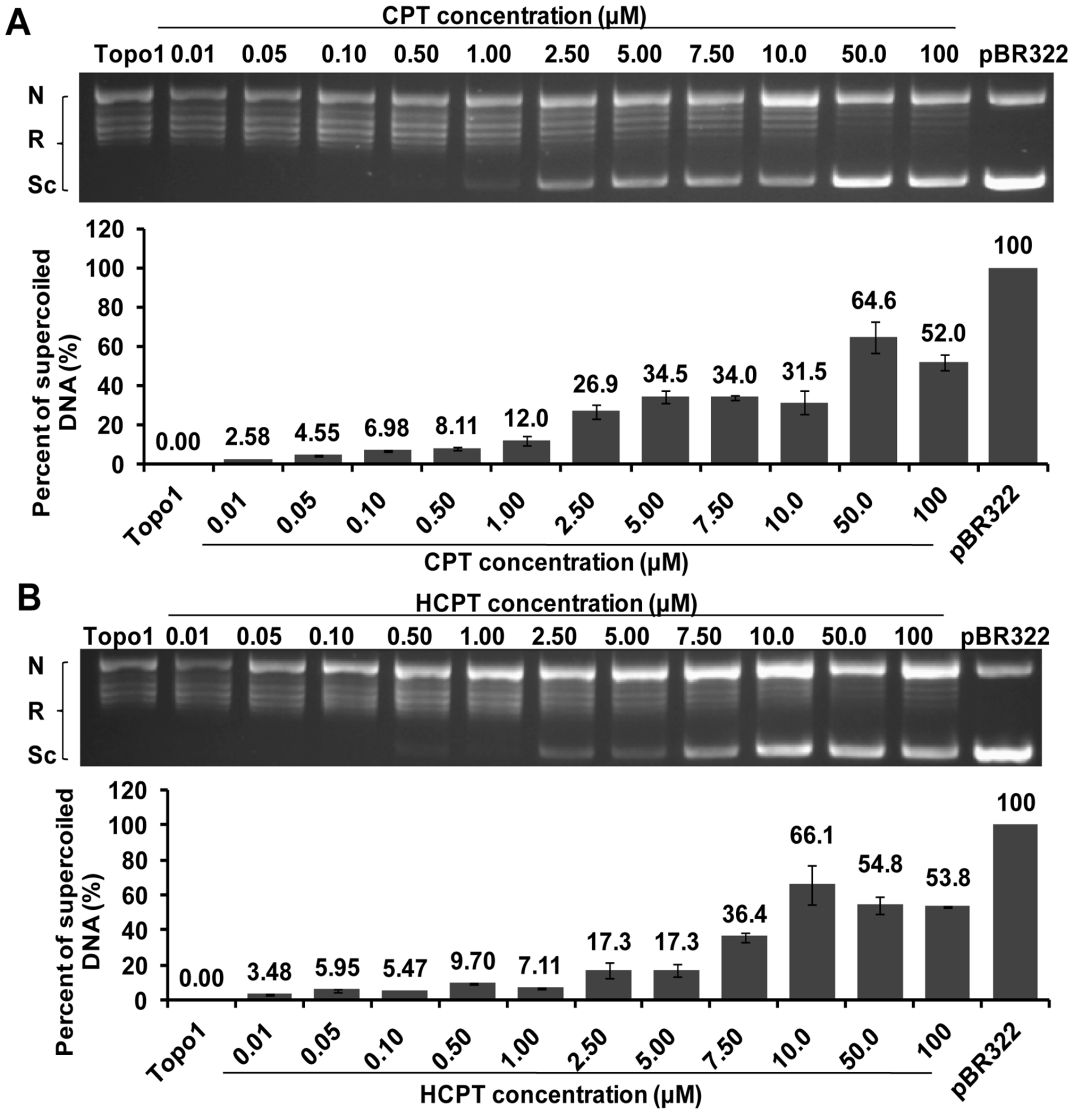
CPT and HCPT inhibited the DNA relaxation activity of *S. exigua* Topo1 extracted from IOZCAS-Spex-II cells. To evaluate the toxicity of CPT or HCPT to *S. exigua* Topo1, an in vitro double-stranded DNA relaxation assay was performed by incubating Topo1 crude extracts from IOZCAS-Spex-II cells with 0.5 µg pBR322 DNA and various concentrations of CPT or HCPT at 26°C for 30 min. The DNA was subsequently assessed by agarose gel electrophoresis, and the DNA bands were densitometrically quantified with Quantity One (Gel Doc XR, Bio-Rad, USA). The experiments were repeated three times. The inhibition rate of the Topo1 enzymatic activity by CPT or HCPT was calculated as the percentage of supercoiled DNA over total pBR322 DNA. Each bar represents the mean ± SD. A: CPT; B: HCPT; Sc: supercoiled DNA; R: relaxed DNA; N: nicked DNA; pBR322: pBR322+0.50% DMSO; Topo1: Topo1+pBR322+0.50% DMSO.

**Figure 6:**
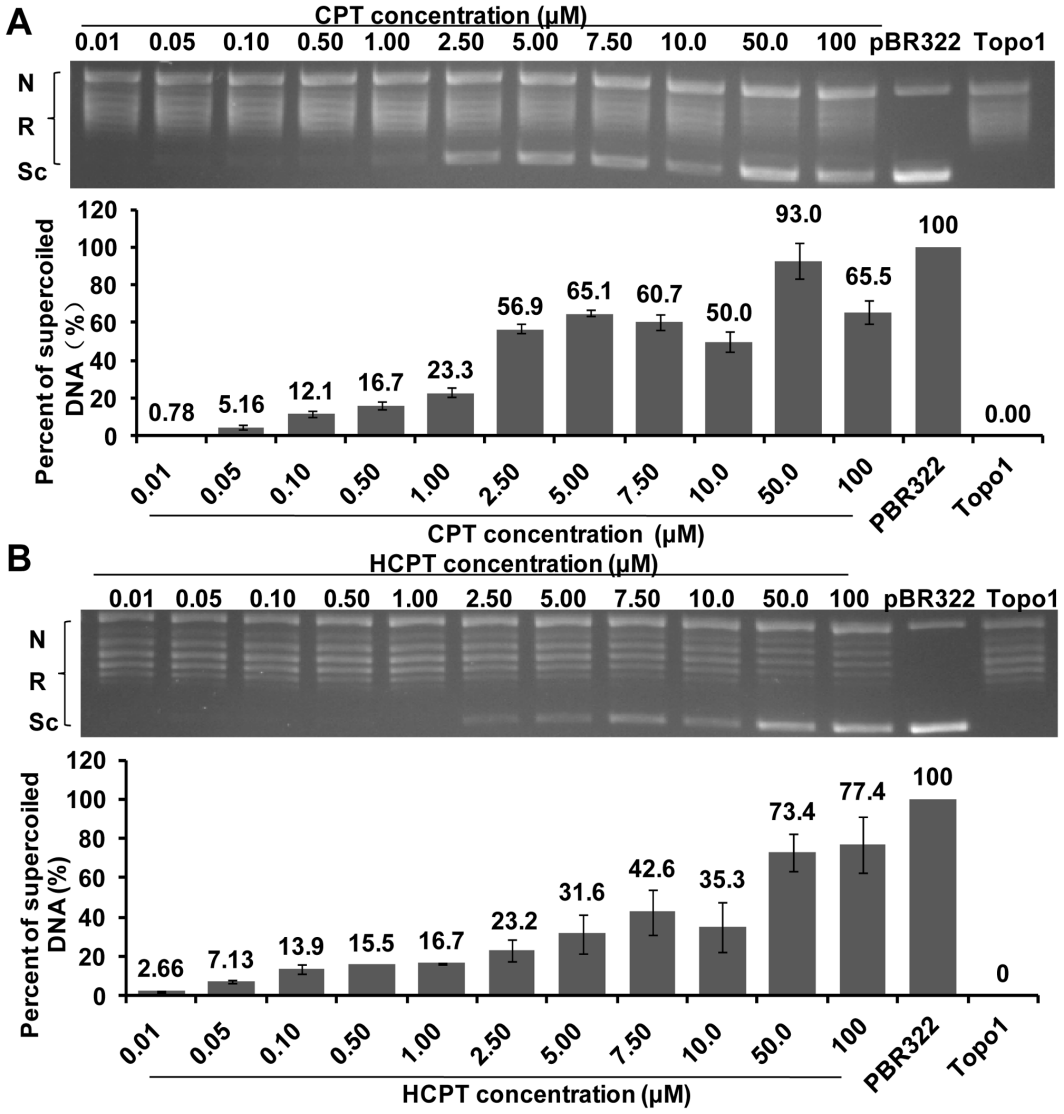
CPT and HCPT inhibited the DNA relaxation activity of truncated Topo1 expressed in *E.coil*. To test the toxicity of CPT or HCPT to *S. exigua* Topo1, the purified recombinant Topo1 protein was incubated with 0.5 µg pBR322 DNA and various concentrations of CPT or HCPT at 26°C for 30 min. The ability of Topo1 to relax DNA in the presence or absence of CPT or HCPT was analyzed by agarose gel electrophoresis, and the DNA bands were densitometrically quantified with Quantity One (Gel Doc XR, Bio-Rad, USA). The experiments were repeated three times. The inhibition rate of the Topo1 enzymatic activity by CPT or HCPT was calculated as the percentage of supercoiled DNA over total pBR322 DNA. Each bar represents the mean ± SD. A: CPT; B: HCPT; Sc: supercoiled DNA; R: relaxed DNA; N: nicked DNA; pBR322: pBR322+0.50% DMSO; Topo1: Topo1+pBR322+0.50% DMSO.

To investigate whether the natural *S. exigua* Topo1 is sensitive to CPT and HCPT treatment as expected, the Topo1 crude extracted from IOZCAS-Spex-II cells was used. 1 U Topo1 enzyme was incubated with the reaction buffer which pre-mixed with different concentrations of CPT or HCPT. The natural Topo1 completely relaxed supercoiled DNA (Figure 5A and 5B, line Topo1), and was susceptible to the treatment of both CPT and HCPT in a similar dose-dependent manner. The two compounds had respective EC_50_ values of 42.5 µM (95% fiducial limits, 20.8–86.9 µM) and 48.9 µM (95% fiducial limits, 11.5–207 µM). As for the recombinant Topo1, a similar dose-dependent inhibitory effect was also observed for both CPT and HCPT (Figure 6A and 6B). HCPT reached the maximum inhibition (Figure 6B, the percent of supercoiled DNA, 73.4%) at 100 µM and CPT at 50 µM (Figure 6A, the percent of supercoiled DNA, 93.0%). The respective EC_50_ values were 4.42 µM (95% fiducial limits, 2.22–8.82 µM) and 15.2 µM (95% fiducial limits, 8.05–28.7 µM), an inhibitory effect that was much higher than that from cell extracts, suggesting some unknown factors may interfere with the activity of CPT and HCPT.

### CPT and HCPT Treatment of IOZCAS-Spex-II Cells Reduced Topo1 Specific Activities

**Figure 7:**
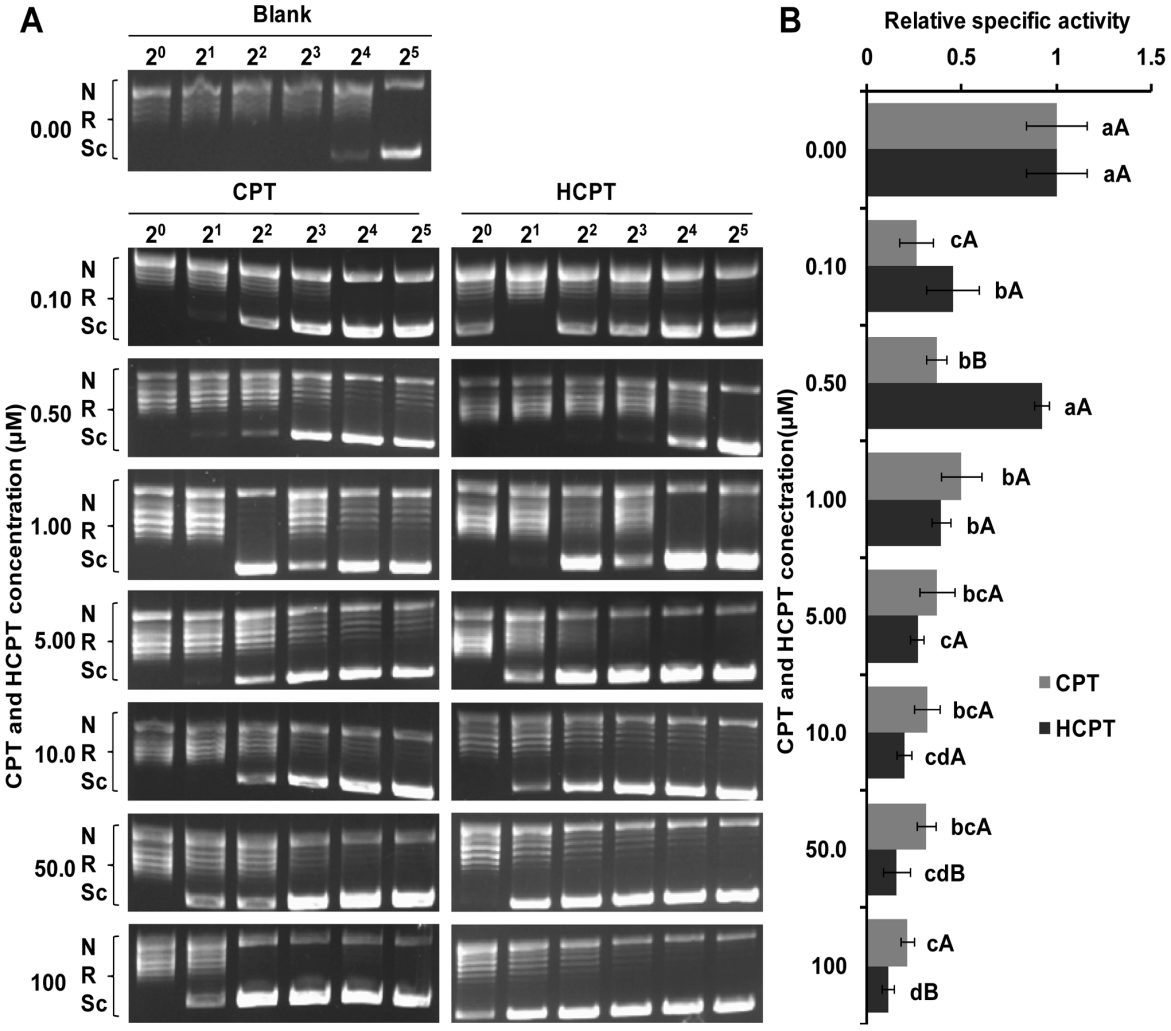
Pre-treatment of IOZCAS-Spex-II cells with CPT and HCPT decreased Topo1 specific activity in a dose-dependent manner. (A) The IOZCAS-Spex-II cells were pretreated with various concentraions of CPT or HCPT for 24 h prior subjected to Topo1 extraction. The CPT and HCPT toxicity was assessed by incubating serial of 2-fold diluted Topo1 extracts with 0.5 ug DNA at at 26°C for 30 min. The DNA relaxation ability was then analyzed by agarose-gel electrophoresis. The picture shown was from a single representative experiment out of three repeats. Sc: supercoiled DNA; R: relaxed DNA; N: nicked DNA; 2^0^, 2^1^, 2^2^, 2^3^, 2^4^, 2^5^: the serial two-fold dilutions; Blank: pretreated with only 0.50% DMSO for 24 h. (B) The relative specific activity of Topo1 was expressed as the ratio of the specific activity of Topo1 from CPT or HCPT treated cells over that from Blank. Each bar represents the mean ± SD. Means with the same letter are not significantly different (Student’s *t*-test, *p*>0.05). Small letters represent the comparison within different dosage scales. Capital letters represent the comparison between CPT (gray) and HCPT (dark).

**Figure 8:**
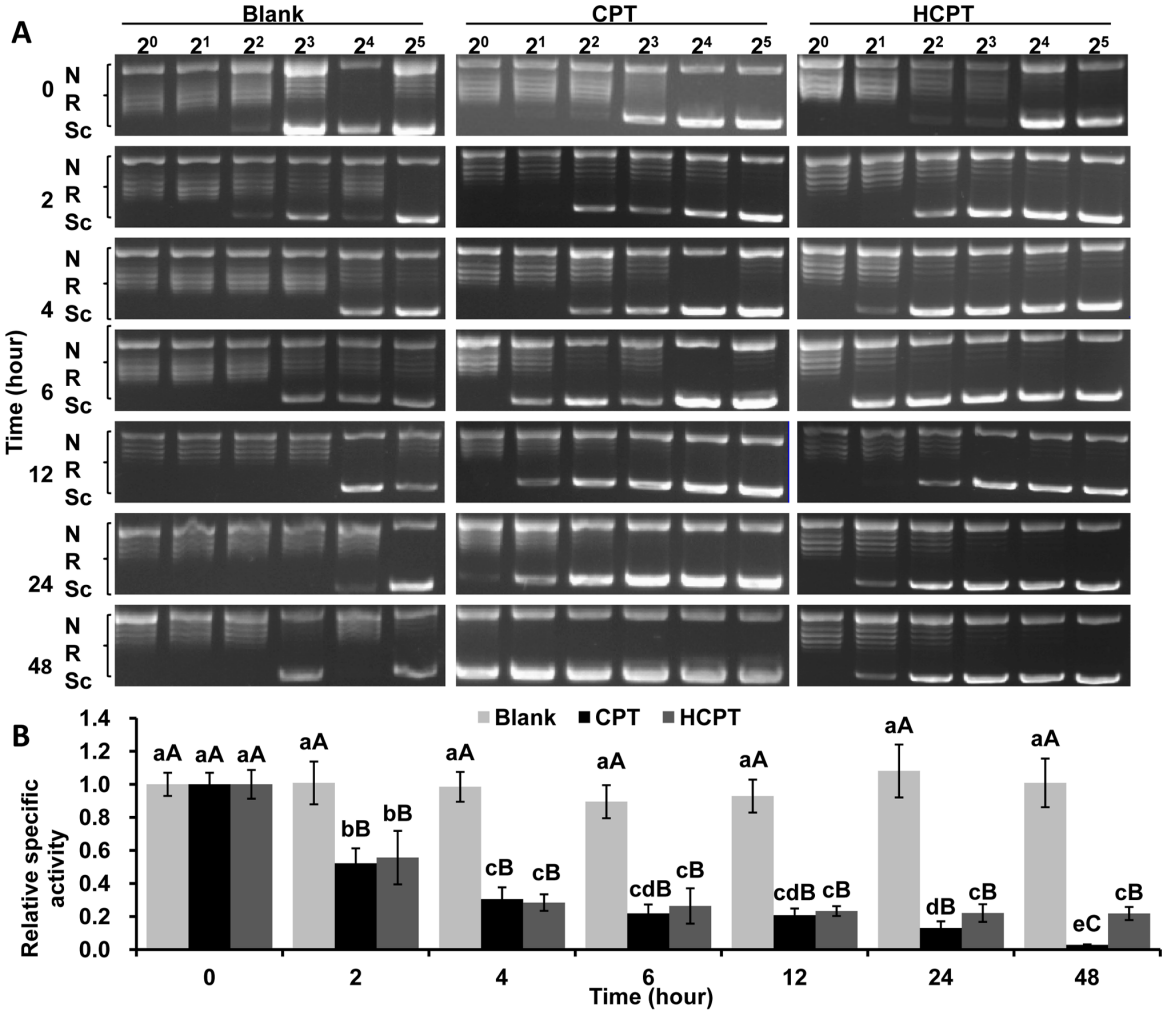
Pre-treatment of IOZCAS-Spex-II cells with CPT and HCPT decreased Topo1 specific activity in a time-dependent manner. (A) The IOZCAS-Spex-II cells were pretreated with 10 uM CPT or HCPT for various times as indicated before being subject to Topo1 extraction. The toxicity to Topo1 was evaluated by incubating serial of 2-fold diluted Topo1 extracts with 0.5 µg DNA at 26°C for 30 min. The DNA relaxation ability was then analyzed by agarose-gel electrophoresis. The picture shown was from a single representative experiment out of three repeats. Sc: supercoiled DNA; R: relaxed DNA; N: nicked DNA; 2^0^, 2^1^, 2^2^, 2^3^, 2^4^, 2^5^: the serial two-fold dilutions; Blank: pretreated with only 0.10% DMSO for various times. (B) The relative specific activity of Topo1 was calculated as the ratio of the specific activity of Blank or CPT or HCPT for various time points over that from Blank or CPT or HCPT for 0 h, respectively. Each bar represents the mean ± SD. Means with the same letter are not significantly different (Student’s *t*-test, *p*>0.05). Small letters represent the comparison within each time point. Capital letters represent the comparisons among the Blank (white), CPT (dark) and HCPT (gray).

To test whether CPT and HCPT treatment can reduce the enzymatic activity of Topo1 *in vivo*, IOZCAS-Spex-II cells were pretreated with different doses of these chemicals for various times. As shown in Figure 7A, Topo1 specific activity dropped significantly in cells when treated with increased concentrations of CPT and HCPT for 24 h with the exception of Topo1 in cells treated with 0.5 µM HCPT (Figure 7B). In contrast, there was no significant change in Topo1 specific activity in the DMSO treated cells for all the test time points (Figure 8A and 8B). In the presence of CPT and HCPT, Topo1 specific activity (the relative specific activity, from 0.03 to 0.52 for CPT; from 0.22 to 0.57 for HCPT) decreased significantly with the increase of incubation time (*p*<0.05). There was no appreciable difference in Topo1 specific activity between CPT and HCPT pretreated cells. Thus, the above observations indicated that CPT/HCPT pretreatment induced a time- and dose-dependent loss of Topo1 enzyme activity in IOZCAS-Spex-II cells.

### Upregulation of Topo1 Gene Expression in IOZCAS-Spex-II Cells Pretreated with CPT and HCPT

**Figure 9:**
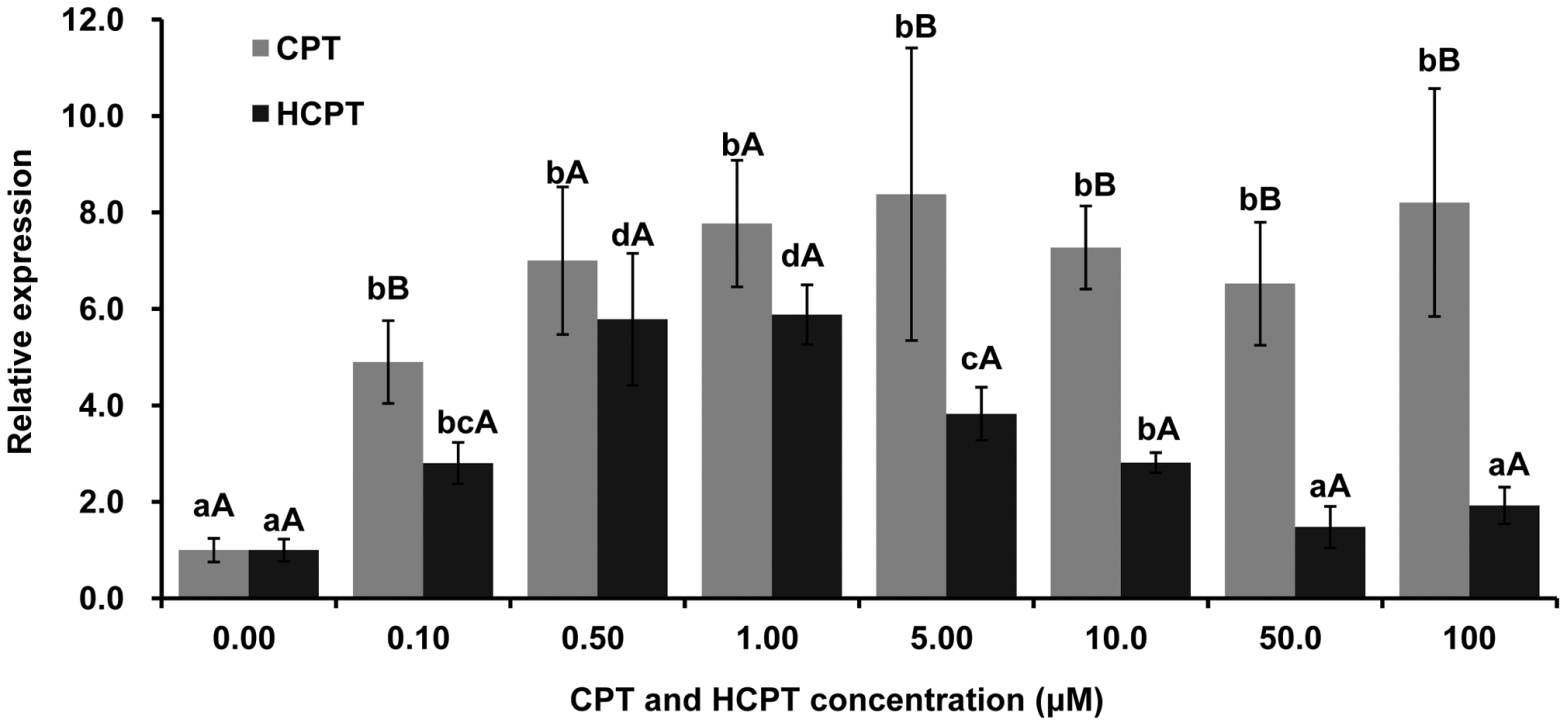
Comparative quantitative real-time PCR analysis of *S. exigua Topo1* gene expression on diverse dosage scales. IOZCAS-Spex-II cells were treated with various concentrations of CPT or HCPT, and harvested at 24 h post treatment prior to being subject to total RNA extraction. The mRNA expression level of *Topo1* was measured by Real time PCR as described in Materials and Methods. *Topo1* gene expression was normalized in reference to the internal control (beta-actin). The relative expression was calculated as the ratio of *Topo1* gene expression from CPT or HCPT pretreated cells over that from without CPT or HCPT treatment (0.00, treated with only 0.50% DMSO). Each bar represents the mean ± SD. Means with the same letter are not significantly different (Student’s *t*-test, *p*>0.05). Small letters represent the comparison within each time point. Capital letters represent the comparison between CPT (dark) and HCPT (gray).

**Figure 10:**
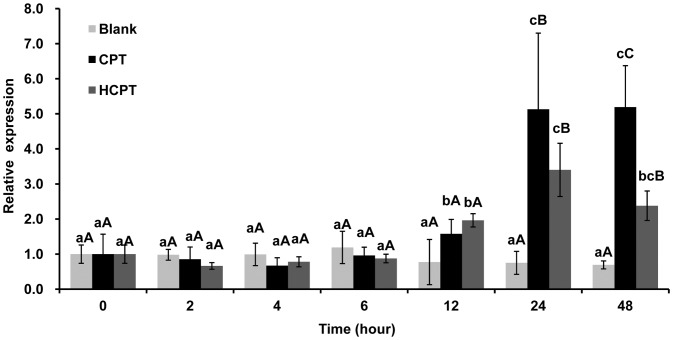
Quantitative real-time PCR analysis of *S. exigua Topo1* gene expression. IOZCAS-Spex-II cells were pretreated with either 0.1% DMSO or 10 µM CPT or HCPT and harvested at different time points post treatment (0, 2, 4, 6, 12, 24 and 48 h) before being subjected to total RNA extraction. The mRNA expression level of Topo1 was measured by Real-time PCR as described in Materials and Methods. *Topo1* gene expression was normalized in reference to the internal control (beta-actin). The relative expression was calculated as the ratio of *Topo1* gene expression of different time points from 0.1% DMSO (Blank) or CPT or HCPT pretreated cells over that of 0 h, respectively. Each bar represents the mean ± SD. Means with the same letter are not significantly different (Student’s *t*-test, *p*>0.05). Small letters represent the comparison within each time point. Capital letters represent the comparisons among the Blank (white), CPT (dark) and HCPT (gray).

To test whether or not the decreased enzymatic activity of Topo1 upon CPT and HCPT pretreatment is due to the reduced *Topo1* gene expression, the mRNA expression of *Topo1* was measured by Real-time PCR with beta-actin as an internal control. Surprisingly, the mRNA expression of *Topo1* was significantly up-regulated in CPT and HCPT treated IOZCAS-Spex-II cells. The *Topo1* gene expression was increased in cells treated with all concentrations of CPT (the relative expression, from 4.90 to 8.37) and HCPT (the relative expression, from 1.48 to 5.88) for 24 h compared to the mock treated cells (Figure 9). HCPT-treated cells showed less *Topo1* expression than CPT-treated cells with all corresponding concentrations. At all the tested time points, there was no significant change in *Topo1* expression in the control group (Figure 10, Blank). However, *Topo1* gene expression was up-regulated at 12, 24 and 48 h in cells treated with 10 µM CPT (1.58–5.19) and HCPT (1.96–2.38).

### Pretreatment of IOZCAS-Spex-II cells with CPT and HCPT Resulted in Reduction in Steady Accumulation of Topo1 Protein

It has been demonstrated that prolonged treatment of human cancer cells with CPT led to down-regulation of Topo1 in a dose- and time-dependent manner due to Topo1 protein degradation and re-localization [30]. Also, the *Topo1* mRNA level in the CPT-resistant cell lines has been found to be reduced, which accordingly reduced cellular Topo1 protein level as measured by western blotting in nuclear extracts [5], [31], [32]. However, it is not always unusual that Topo1 proteins and CPT-stabilized Topo1 cleavable complexes are not altered accordingly in some CPT-resistant cancer cells, suggesting that the CPT cytotoxicity may depend on cell types for other unknown mechanisms [33].

To determine whether CPT and HCPT treatment altered the expression of the Topo1 protein in IOZCAS-Spex-II cells, a ployconal antibody (anti-Topo70) against beet armyworm Topo1 was produced by immunizing rabbits with purified recombinant Topo1 (Topo70) by Immunosoft Ltd. (Zhoushan, China). This polyclonal antibody strongly reacted with the recombinant GST-Topo70 fusion protein. Detection of native beet armyworm Topo1 from cell crude extracts revealed a band of 130 kD, which was different from the predicted size of 108 kD, suggesting further posttranslational modifications. The specificity was further supported by a competition assay. Pre-incubation of the antibody to purified GST-Topo70 blocked the recognition of the 130 kD band, suggesting that the 130-kD polypeptide is the beet armyworm Topo1.

**Figure 11:**
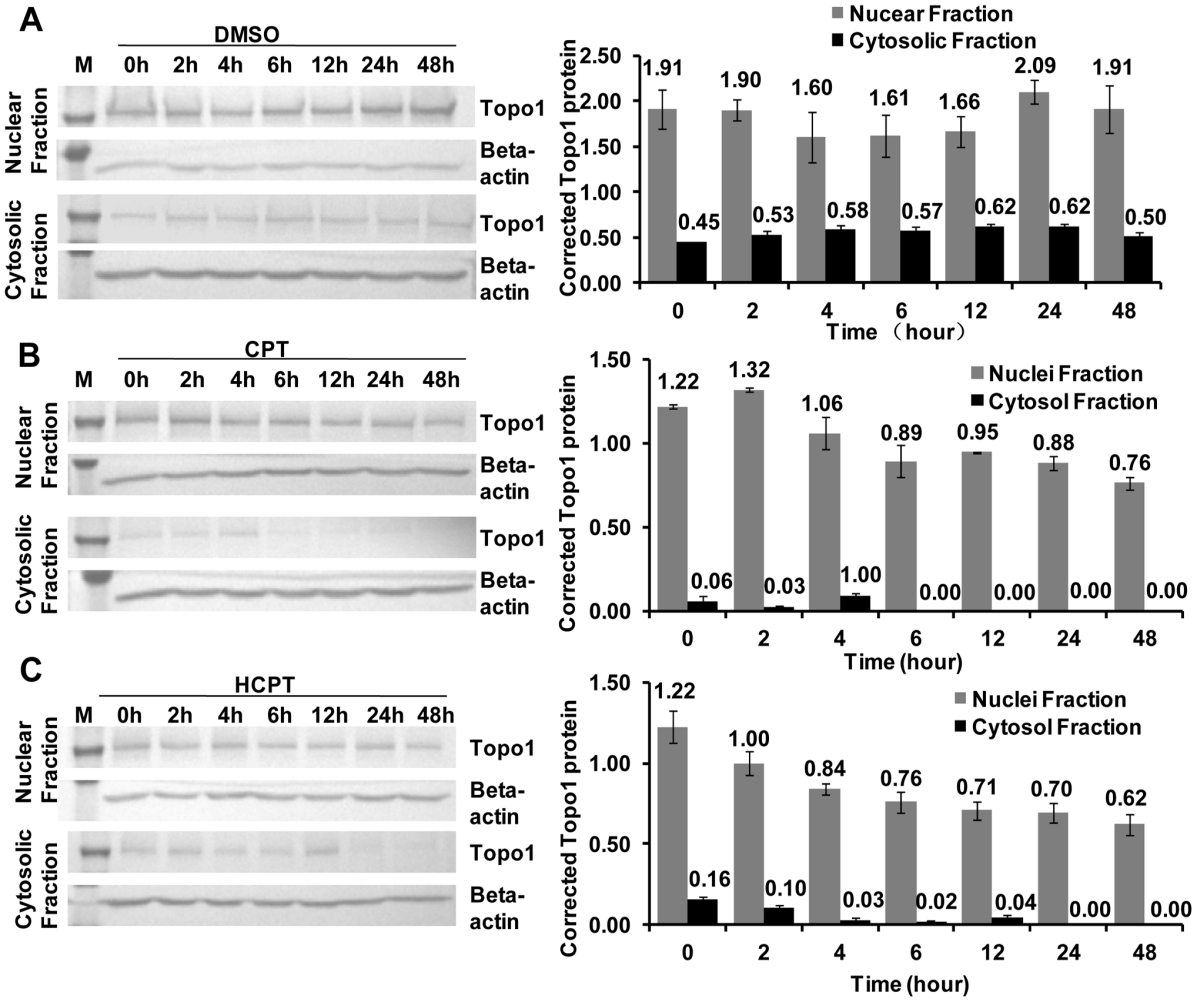
CPT and HCPT treatment induced Topo1 protein down-regulation. IOZCAS-Spex-II cells pretreated with 0.1% DMSO (A), or 10 µM CPT (B) or HCPT (C) were harvested and lysed at different time points post treatment (0, 2, 4, 6, 12, 24 and 48 h). The cytosolic and nuclear fractions of cells were prepared as described in Materials and Methods. Equal amounts of protein from each fraction (40 µg for nuclear fraction and 80 µg for cytosolic fraction) were subject to electrophoresis in denaturing gels, and immunoblotted with indicated antibodies. Beta-actin was used as a loading control. The protein bands were densitometrically quantified with Quantity One (Gel Doc XR, Bio-Rad, USA). The corrected Topo1 protein was calculated as the ratio of nuclear (gray) or cytosolic (dark) fraction over beta-actin. Each bar represents the mean ±SD.

We next tested the expression level of Topo1 in the CPT and HCPT pretreated cells. As shown in Figure 10, both 10 µM CPT (Figure 11B) and HCPT (Figure 11C) induced a certain Topo1 protein reduction in nuclear fraction during the time course compared to that extracted from cells treated with the 0.1% DMSO (Figure 11A). The down-regulation of Topo1 protein showed a time-dependent manner. Interestingly, the 130-kD polypeptide in could still be detected in cytosolic fraction, and exhibited a decreased in a time-dependent pattern on CPT and HCPT treatment.

## Discussion

During the long adaptive evolution, Topo1s in plants and animals have shared identical spatial structures and biological functions among different species, and are particularly highly conserved in both the core and carboxyl domains, where all the active sites are located. The *Topo1* gene isolated from *S. exigua* in this study contains an open reading frame 2790 bp encoding a polypeptide of 930 amino acids. The deduced protein consists of four major regions like other eukaryotic Topo1: the NH_2_-terminal, core, linker and carboxyl domains, with the conserved four active-site residues (R488, K532, R590 and H632) and the catalytic Y723. The amino acid polymorphism of Topo1s at the sites directly/indirectly related to CPT binding illustrates the important footprint of coevolution between CPT-producing plants and animals [22], [34], [35]. As shown in Figure 3, the amino acid polymorphism of Topo1s is detectable and widespread in different unrelated species, offering themselves a chance of CPT resistance or sensitivity [25]. Sikikantarmas et al. reported the survival strategy of CPT-producing plants against the CPT self-toxicity through amino acid substitutions in *Topo1* compared to *H*. *sapiens* [35]. Three amino acid substitutions, N421K, L530I and N722S existing naturally in Topo1 of CPT-producing plants, are related to CPT-resistance. In particular, the substitution N722S is identical to that observed in CPT-resistant cancer cell lines. Additionally, more than ten mutations have been reported to be associated with resistance to CPT and its derivatives in human cancer cell lines established from screening Topo1 mutants stepwisely with CPT [24], [26], [35]. These findings reveal a very exciting example to understand the coevolution not only between the CPT biosynthetic pathway and self-resistance mechanism in CPT-producing plants, but also between plants and their natural enemies including insects and human. In nine reported insect Topo1s, four amino acid substitutions were also found at the sites involved in DNA binding or structural interaction with CPT. The effect of these four amino acid polymorphisms on CPT-sensitivity/resistance in insects is worthy of further studies, which may be useful for designing better CPT analogues.

CPT and HCPT are poisonous to a broad spectrum of Topo1s from different eukaryotic species with no exception to *S*. *exigua* Topo1. Generally, CPT derivatives are expected to have improved solubility, stability and long lifetime of the lactone form. HCPT is formed by hydroxy substitution at position 10 of CPT, which also is a stronger Topo1 inhibitor than CPT in many studies [19]. Modifications at the specified positions help alter the equilibrium of the carboxylate/lactone form of the E ring and are directly associated with drug toxicity [36], [37]. CPT and its derivatives with a closed E ring are more effective than those with an open E ring [38]. Our results showed that both CPT and HCPT exhibited a dose- and time- dependent effect on Topo1 enzyme activity. Because the lactone form of the E ring in HCPT is preferentially pH-dependent, HCPT showed lower inhibitory effects *in vitro* than CPT in a reaction buffer of pH 7.5 (Figures 5 and 6). In addition, the *Topo1* gene expression in the HCPT-treated cells was lower than that in the CPT-treated sample for 24 h treatment (Figure 9, Figure 10). These results suggest that CPT is more toxic to *S*. *exigua* Topo1 than HCPT. The difference of CPT and HCPT toxicity on the *S*. *exigua* Topo1 requires further investigation in the future.

The crucial role of Topo1 for cell survival and differentiation requests a strict regulation of its expression. Upon CPT treatment, Topo1 is the first protein to be regulated both in redistribution and in Topo1 protein level in a single clone H1299-cherry [30]. Topo1 intensity in the nucleoli dropped in less than 2 min and it accumulated in the cytoplasm up to 5 hours in a CPT dose-dependent manner. Topo1 protein was finally degraded into detectable fragments of ∼40 kD in the nucleus and then Topo1 fragments exited the nucleus into cytoplasm [30]. In this paper, the Topo1 enzyme activities and protein levels in drug-treated cells decreased despite the improved *Topo1* mRNA levels (Figures 7, 8, 9, 10 and 11). Although the degraded fragments of Topo1 proteins were not detected both in cytosolic and nuclear fractions, the down-regulation of Topo1 protein explained well the decreased enzymatic activity. Because CPT/HCPT-treated cells need a large quantity of Topo1 proteins to resolve DNA topological problems in DNA repair or other apoptosis-associated DNA gene expression [39], [40], the *Topo1* mRNA level was accordingly increased to ensure that enough Topo1 protein is synthesized for the efficient DNA repair or other gene expression in response to Topo1 down-regulation induced by CPT and HCPT in IOZCAS-Spex-II cells.

In summary, our studies suggest that *S. exigua* Topo1 can be inactivated in the presence of CPT and HCPT with time- and dose-effect, which provides a suitable experimental model for further studies focused on exploring derivates of CPT as insecticides.

## Materials and Methods

### Culture of Insect Cells and CPT and HCPT Preparation

IOZCAS-Spex-II cells, a cell line derived from *S. exigua* mid-gut fat bodies, were provided by the Institute of Zoology of Chinese Academy of Science (Beijing, China), and maintained at 27°C in Grace’s insect medium supplemented with 10% fetal bovine serum (FBS, Heat-inactivated, Invitrogen, CA, USA) in T25 cm^2^ tissue culture flasks (Corning, USA). The cultures were sub-cultured every 6 days.

CPT (99.17%) and HCPT (99.44%) were purchased from Sichuan Nanbu Chenxin Technology Co., Sichuan, China, and dissolved in 100% dimethyl sulfoxide (DMSO).

### Isolation of *S. exigua* Topo1 Gene

Total RNA was isolated from IOZCAS-Spex-II cells with RNeasy® Mini kit (Qiagen GmbH, Germany). cDNA was synthesized with 1 µL total RNA using the Omniscript® RT Kit (QIAGEN GmbH, Germany) following the manufacturer’s instructions. The degenerate and specific primers listed in Table 2 were designed to target the conserved regions obtained from the multiple alignment analysis of Topo1s from different species with DNAMAN (Lynnon Biosof, USA).

**Table 2:** Primers used in this study.

Enzyme	Total Protein (µg)	Total Activity (U)	Specific Activity (U mg^−1^ pro)	Yield (%)
Crude Extracts	372	128,000	344,000	100
Fraction #2	17.8	32,000	1,797,600	4.78

Y = C or T; R = A or G; H = A, C or T; M = A or C; W = A or T; D = A, G or T; N = A, T, C or G.

a Bases underlined are the restriction site for *BamH* I.

b Bases underlined are the restriction site for *Sal* I.

The PCR amplification was conducted in a 25 µL reaction volume containing 200 µM dNTPs, 1×PCR buffer and 1U Taq DNA polymerase (Takara, Dalian, China), with an initial denaturation step of 94°C for 5 min and followed by 30 cycles of 94°C for 30 s, 56°C for 30 s and 72°C for 1 min. A final step for 10 min at 72°C was used to fully extend the amplicons. The target PCR product was purified using QIAquick Gel Extraction Kit (QIAGEN) and ligated into pGEM-T easy vector (Promega, USA) for sequencing at Sangon Bio-engineering (Shanghai, China). The 5′and 3′ ends were amplified with gene-specific primers listed in Table 1 and the adaptor primers were provided by the kits of 5′-Full RACE Core Set Ver.2.0 and 3′-Full RACE Core Set Ver.2.0 (Takara, Dalian, China). The full-length cDNA was assembled by overlapping all the amplified fragments, and deposited in National Center for Biotechnology Information (NCBI) with GenBank ID: JN258956.

### Phylogenetic Analysis of Topo1s

The protein sequences of Topo1s retrieved from National Center for Biotechnology Information (NCBI) and KAIKObase were aligned with ClustalX 1.83 by using standard parameters and then rendered with ESPript 2.2 (http://espript.ibcp.fr/ESPript/cgi-bin/ESPript.cgi). A phylogenic tree was constructed with MEGA version 5.0 program using the neighbor-joining method with 1,000 replicates [41], [42].

### Recombinant Protein Expression and Purification

The Topo1 encoding the residues 337–939 (Topo70) was amplified by PCR with the specific primers and cloned into the vector pGEX-4T-1 at the restriction sites of *BamH* I and *Sal* I (Table 2) to generate plasmid pGST-Topo70. *E.coil* BL21 (DE3) cells were transformed with pGST-Topo70 or pGEX-4T-1 and grown at 37°C. Exponentially growing bacteria in LB medium including 100 µg/mL ampicillin (OD_600_ = 0.6) were treated with 0.6 mM isoproyl-1-thio-β-D-galactopyranoside (IPTG) to induce target protein expression for 4 hours at 30°C.

The cultures were harvested by centrifugation at 8,000 rpm for 5 min, washed with the PBS buffer (pH 7.4, 140 mM NaCl, 2.7 mM KCl, 10 mM Na_2_HPO_4_, 1.8 mM KH_2_PO_4_) twice, and resuspended in lysis buffer (50 mM Tris–HCl, pH7.5, 250 mM KCl, 0.5% Triton X-100, 1 mM DTT, 2 mM EDTA and 1 mM PMSF) with a final concentration of 250 µg/mL lysozyme on ice for 15 min. The samples were then spun at 15,000×g for 30 min to remove cell debris and insoluble materials. The Topo1 proteins in the collected supernatants were purified with the GSTrap 4B columns (GE Healthcare, UK) following the manufacturer’s instructions. The recombinant Topo1 was eluted to seven fractions with an equal volume of 500 µL. The concentrations of the protein were determined by Bradford methods [43] and the purified Topo1 was confirmed by SDS-PAGE. The recombinant Topo1 was kept in 50% glycerol with aliquots at −80°C.

### The Activity Unit and Specific Activity of Topo1 Expressed in *E.coil*

The DNA topoisomerase-1 relaxation assay was performed according to the procedures as previously reported with some modifications [44]. One unit (U) of Topo1 activity was defined as the amount of enzyme activity that will relax 0.5 µg pBR322 DNA fully at 26°C in 30 min. The specific activity was expressed in units of enzyme per milligram protein (U mg^−1^ pro).

In the absence of Mg^2+^ for avoidance from interference of prokaryotic Topo1, protein samples were diluted by two-folds and each dilution was incubated with the reaction buffer (150 mM KCl, 10 mM Tris-HCl (pH 7.5), 1 mM DTT, 1 mM EDTA, 0.1 mg/mL BSA) and 25 ng/µL (final concentration) plasmid DNA in 20 µL volumes under 26°C for 30 min to ensure the maximum relaxation of the DNA. The reaction was terminated by adding proteinase K (250 µg/mL) and 0.5% SDS (final concentration), and then incubated at 50°C for additional 30 min to remove extra proteins from DNA. Subsequently, three different forms of DNA (the supercoiled, relaxed and nicked form) were separated by electrophoresis in 1% agarose gel.

### Inhibition of CPT and HCPT on the Activity of Recombinant and Natural Topo1s

Both recombinant and natural Topo1s were used for the toxicity analysis. The natural Topo1 was extracted from IOZCAS-Spex-II cells according to the modified method [45]. After removal of the insect medium, insect cells were harvested by centrifugation at 400×g for 5 min and washed with PBS three times. The cell pellet was resuspended in 180 µL lysis buffer (pH 7.5, 50 mM KCl, 10 mM Tris-HCl, 15 mM DTT, 2 mM MgCl_2_, 1% Triton X-100, 1 mM PMSF) by vigorously shaking for 5 min on ice. The nuclei was collected by sedimentation of 600×g for 10 min and then stirred in 120 µL resuspension buffer (pH 7.5, 50 mM KCl, 10 mM Tris-HCl, 2 mM MgCl_2_) containing 15 mM DTT, 1 mM PMSF. The pelleted nuclei were spun at 600×g for 10 min. The collected nuclei were then pelleted in 50 µL resuspension buffer containing 25 mM DTT, 1 mM PMSF and 10 mM EDTA. 50 µL of 2×nuclear extraction buffer (2M NaCl, 80 mM Tris-HCl (pH 7.5), 20% glycerol, 2mM EDTA) was added to lyse the nuclei for 15 min. Crude extracts were collected by discarding the precipitated nucleic acids in PEG buffer (1M NaCl, 18% PEG, 10% glycerol) by centrifugating at 10,000×g for 30 min.

The enzymatic activity of Topo1s was determined according to methods mentioned above. Both 1U of recombinant and natural Topo1s were incubated with various concentrations (from 0.01 to 100 µM) of CPT and HCPT in a total of 20 µL reaction buffer under the same reaction condition. DNA bands were visualized with a digital UV transilluminator and quantified with Quantity one (Gel Doc XR, Bio-Rad, USA). The inhibitory rate (%) of the enzymatic activity by CPT and HCPT was calculated as the percentage of supercoiled over total pBR322 DNA. The EC_50_ value was calculated by using Probit analysis with DPS (v 9.5, Zhejiang University, China) [46].

### Effects of CPT and HCPT Pre-treatment on the Specific Activity of Topo1 in *S. exigua*

Two series of experiments were conducted to analyze the effects of CPT and HCPT treatment on the catalytic activity of *S. exigua* Topo1. The first series examined changes of Topo1 activity caused by 10 µM CPT and HCPT pre-treatment for different times (0, 2, 4, 6, 12, 24 and 48 h). The second series of experiments examined the effects on the Topo1 specific activity with different concentrations (0.10, 0.50, 1.00, 5.00, 10.0, 50.0, 100 µM) of CPT and HCPT treatment for 24 h.

In all experiments, IOZCAS-Spex-II cells were pretreated with CPT and HCPT according to the procedure described in our previous studies [16]. The Topo1 was extracted from IOZCAS-Spex-II cells, and the specific activity of the nuclear extract in each sample was determined using the method described above.

### Real-time PCR Analysis

The *Topo1* mRNA level in CPT and HCPT treated cells was measured by Real-time PCR. The reactions were carried out in strip tubes with caps (0.2 mL, AXYGEN, USA) in a total volume of 20 µL, including 10 µL SYBR Premix Ex Taq™ (Takara, Dalian, China), 0.4 µL forward primer and reverse primer (10 µM), 0.4 µL ROX reference dye II, 2.0 µL reverse transcribed cDNA and 6.8 µL H_2_O. The procedure was carried out with two steps as follows: 30 sec at 95°C; 40 cycles of 5 sec at 95°C and 30 sec at 60°C. Gene expression was normalized to beta-actin with 2^-△△Ct^ method [47]. Data was analyzed with 7500 software V 2.0.3 (Applied Biosystems, CA, USA).

### Western Blot Analysis

IOZCAS-Spex-II cells were pretreated with 10 µM CPT and HCPT for 24 h prior to fractionation with the method as described by Fu et al. [48]. Total protein concentration was determined by Bradford methods [43]. The equal amount of samples were separated by 8% SDS-PAGE, transferred to nitrocellulose membranes and blocked in blocking buffer containing 5% non-fat milk (25.0 mM Tris, 150 mM NaCl, 0.1% Tween20, pH 7.5) for 1 h at the room temperature. The blots were then probed with rabbit polyclonal antibodies to topoisomerase I produced with the recombinant Topo1 (Topo70) by Immunosoft Ltd. (1∶5000, Zhoushan, China) followed by mouse anti-rabbit IgG-HRP antibody (1∶10000, R&D Systems, Inc., MN, USA). For loading control, beta-actin was probed with mouse anti-actin monoclonal antibody (Abcam Ltd., Hongkong) and secondary primary, anti-mouse IgG-HRP antibody (R&D Systems, Inc., MN, USA). The signals were detected with a DAB detection kit (Boster, Wuhan, China).

### Statistical Analysis

All experiments were repeated at least three times and results were expressed as mean ± standard deviation (SD). Statistical analysis was carried out by one-way ANOVA followed by Student’s *t*-test with a statistically significant value of *p*<0.05.
